# A panel of three serum microRNA can be used as potential diagnostic biomarkers for nasopharyngeal carcinoma

**DOI:** 10.1002/jcla.24194

**Published:** 2022-01-14

**Authors:** Rongkang Li, Chong Lu, Weiqiang Yang, Yaqi Zhou, Jiatao Zhong, Xuan Chen, Xinji Li, Guocheng Huang, Xiqi Peng, Kaihao Liu, Chunduo Zhang, Hongyi Hu, Yongqing Lai

**Affiliations:** ^1^ Department of Urology Guangdong and Shenzhen Key Laboratory of Male Reproductive Medicine and Genetics Peking University Shenzhen Hospital Clinical College of Anhui Medical University Shenzhen China; ^2^ The fifth Clinical Medical College of Anhui Medical University Hefei China; ^3^ Department of Otorhinolaryngology Peking University Shenzhen Hospital Shenzhen China; ^4^ Shantou University Medical College Shantou China

**Keywords:** biomarkers, microRNA, nasopharyngeal carcinoma

## Abstract

**Background:**

Nasopharyngeal carcinoma is cancer with unique epidemiological characteristics, showing obvious ethnicity, gender, and geographical prevalence. More and more evidence shows that microRNAs are stable in serum and are specific to different tumor types. Therefore, miRNA is a new non‐invasive biomarker for cancer detection.

**Methods:**

The experiment is divided into three stages, namely, the screening stage, the training stage, and the verification stage. We took 54 patients with nasopharyngeal carcinoma and 108 healthy controls as the research objects. We use the receiver‐operating characteristic (ROC) curve and area under the ROC curve (AUC) to evaluate the diagnostic value of miRNA. Finally, a three‐miRNA panel with high diagnostic efficiency was constructed. In addition, we conducted biological information analysis of these miRNAs to explore their functions.

**Results:**

In NPC patients, the expression of five serum miRNAs (miR‐29c‐3p, miR‐143‐5p, miR‐150‐5p, miR‐145‐3p, and miR‐205‐5p) is significantly dysregulated. Among them, the diagnostic value of these three miRNAs (miR‐29c‐3p, AUC = 0.702; miR‐143‐5p, AUC = 0.733; and miR‐205‐5p, AUC = 787) is more prominent. The diagnostic panel constructed by them has a higher diagnostic value (AUC = 0.902). Through the analysis of the TCGA data set, the target gene of the three‐miRNA panel may be KLF7, NRG1, SH3BGRL2, and SYNPO2.

**Conclusion:**

The three‐miRNA panel (miR‐29c‐3p, miR‐143‐5p, and miR‐205‐5p) may become a novel non‐invasive biological marker for nasopharyngeal cancer screening.

## Graphical abstract

Our study showed that a panel of three serum microRNAs may be performed as a novel and non‐invasive biomarker to diagnose nasopharyngeal carcinoma, with AUC = 0.902. And the development of nasopharyngeal carcinoma may relate to the three‐miRNA panel, the further study was needed to do.

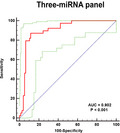

## 1 INTRODUCTION

The distribution of nasopharyngeal carcinoma (NPC) shows obvious ethnicity, gender, and geographical prevalence. Nasopharyngeal cancer has the highest incidence rate in Southeast Asia, such as Singapore, Maldives, and Indonesia with an incidence rate of 7 per 100,000.^1^ Generally, the prognosis of women is better than that of men.^2^, ^3^ So, it is a kind of cancer with unique epidemiological characteristics.^1^ According to research, during 2000–2007, the 5‐year survival rate of European adult NPC was only 49%.^4^ The current diagnosis method is a primary nasopharyngeal tumor biopsy guided by an endoscope. This is an invasive test with poor patient compliance, so it is not suitable for screening. Currently, the commonly used screening methods are plasma EBV DNA and primer/probe analysis for the BamHI‐W region of the EBV genome. However, this examination needs to be performed in duplicate and at least 4 weeks apart.^5^ In addition, the role of EBV in keratinizing cancer is not obvious. In summary, we urgently need a new, non‐invasive, and more reliable screening method.

MicroRNAs (miRNAs) are short (19–22 nucleotides) non‐coding and highly conserved single‐stranded RNAs that can regulate gene expression after transcription.^6^ More and more evidence shows that the expression profile of microRNA can distinguish normal and tumor tissues and is specific to different tumor types.^7^ Studies have found that miRNAs are stably present in serum, which makes it possible to screen for cancer through circulating miRNA analysis.^8^ In summary, the expression pattern of human plasma miRNAs may have the potential to diagnose certain types of cancer.

Our purpose of this study is to find a novel biomarker for the screening of NPC. In this study, we used real‐time quantitative polymerase chain reaction (qRT‐PCR) to find a set of plasma miRNAs, which can be used as a novel biomarker for the screening of NPC. In this experiment, a three‐phase study was used to identify miRNAs with diagnostic value and construct an efficient diagnostic panel. In addition, we also analyzed the biological information of these miRNAs.

## 2 MATERIAL AND METHODS

### 2.1 Participants and ethics statement

All patients were diagnosed as NPC based on histopathological evaluation and did not receive any treatment before sample collection. All these patients have primary tumors and no other metastatic tumors. From November 2017 to May 2021, Peking University Shenzhen Hospital recruited 162 participants, Including 54 NPC patients and 108 healthy controls (HCs). Healthy controls (HCs) are healthy volunteers with no history of cancer, acute or chronic diseases. Every participant fully understood and signed the informed consent form, and the subjects' serum sample collection process also follows the relevant regulations set by the committee. This study was approved and reviewed by the Ethics Committee of Peking University Shenzhen Hospital.

### 2.2 Research design

We conducted a three‐phase study, as shown in Figure S1. Select differentially expressed miRNAs as candidate biomarkers from GSE32960 published on the Gene Expression Omnibus.^9^ In the next step, to determine these candidate biomarkers, we conducted a testing phase and verification phase study. First, in the screening phase, we selected 10 differentially expressed miRNAs, which were related to NPC (Table S1 lists the corresponding reference abstracts of the 10 candidate miRNAs). The primer sequences of these miRNAs are listed in Table S3. Then, in the testing phase, we randomly select 15 serum samples from NPC patients and 30 serum samples from HCs, analyze the expression profiles and diagnostic capabilities of these miRNAs. Finally, focus on verification in the verification phase, and backward stepwise logistic regression analysis is used to construct the panel with the highest diagnostic ability. And there was no significant difference in each group.

### 2.3 Collect serum samples and extract RNA

All participants did not receive any treatment before taking serum samples; first, 2‐ul miR‐54 (cel‐miR‐54‐5p) (10 nm/L, RiboBio) was added to each sample. The purpose of this is to allow it to be used as an internal reference for the RT‐qPCR process and to normalize the variability in the extraction process. Then, total RNA was extracted from serum samples using TRIzol LS isolation kit (Thermo Fisher Scientific) and measured RNA concentration and purity with NanoDrop 2000c spectrophotometer (Thermo Scientific).

### 2.4 Quantitative reverse transcription‐polymerase chain reaction (RT‐qPCR)

To detect the expression level of these miRNAs, the reverse transcription‐specific primers of the Bulge‐LoopmiRNA qRT‐PCR primer set (RiboBio) were used to amplify miRNAs. Then, Taqman probe was used to perform RT‐qPCR on the LightCycler480 Real‐Time PCR system (Roche Diagnostics). The qPCR reaction was performed at 95°C for 20 s, 95°C for 10 s, 60°C for 20 s, and 70°C for 10 s, with 40 cycles. Finally, the 2^−ΔΔCq^ method was used to analyze the relative expression level of target miRNA.^10^

### 2.5 Statistical analysis

The samples of participants in the experiment are divided into a testing phase and a verification phase. The Chi‐squared test for categorical variables or the Student's *t* test for continuous variables was used to test the differences in the expression levels of miRNAs between the NPC and HCs samples. To perform multiple comparisons between different independent stages, we used Kruskal‐Wallis test or Mann‐Whitney test. Receiver‐operating characteristic (ROC) curves and the area under the ROC curve (AUC) were used to evaluate the specificity, diagnostic ability, and sensitivity of each miRNA. Then, the Youden index (calculated as J = Sensitivity + Specificity − 1) was used to determine the most effective diagnostic panel with the highest sensitivity and specificity. SPSS software (SPSS 20.0 Inc), GraphPad Prism 8 (GraphPadSoftware Inc), and Medcalc (Version 19) were used to analyze all data. The clinical characteristics between different groups are expressed in percentage or continuous variable as the means ± SD or number (percentage). The *p* value is considered statistically significant, only when it is less than 0.05.

### 2.6 Bioinformatic analysis

To identify the function of candidate miRNAs in NPC, a series of miRNA targets were predicted in miRWalk2.0 (http://zmf.umm.uni‐heidelberg.de/mirwalk2), and miRWalk is used to predict and experimentally validate miRNA‐target interactions.^11^ Enrichr database (http://amp.pharm.mssm.edu/Enrichr/) was used to perform enrichment analysis and to perform functional annotation on the predicted target gene.^12^ This study used gene ontology annotation (GO) and Kyoto Encyclopedia of Genome (KEGG) approach analysis. Besides, we used OncoLnc (http://www.oncolnc.org/) to generate Kaplan‐Meier survival curves and correlate TCGA survival data with the expression levels of miR‐29c‐3p and miR‐143‐5p and miR‐205‐5p.^13^

## 3 RESULTS

### 3.1 Participants' clinical and demographic characteristics

This experiment involves 54 NPC patients and 108 healthy controls, a total of 162 people. All NPC patients are confirmed by the TNM staging system, and the histological classification is based on the standards of the World Health Organization. All these patients have primary tumors and no other metastatic tumors. All healthy controls had no history of cancer or other diseases. The HCs and NPC patients were divided into a testing phase and a validation phase. The demographic and clinical characteristics of the testing phase and the validation phase are listed in Table 1. Among the two stages, there was no significant difference between NPC and HC both in age and in gender. Parameters were shown as numbers (percentage). Statistical contrast was exerted through the Wilcoxon‐Mann‐Whitney test.

**TABLE 1:** Demographic manifestation of 162 participants (NPC patients and HC)

	Testing phase (*n* = 45)		*p*	Validation phase (*n* = 117)		*p*
	NPC	HC		NPC	HC	
Total number	15	30		39	78	
Age at diagnosis	40.6 ± 11.6	47.4 ± 18.3	0.20	47.5 ± 11.1	51.4 ± 17.9	0.21
Gender			1.00			0.90
Male	8 (53.3%)	16 (53.4%)		21 (53.8%)	41 (52.6%)	
Female	7 (46.7%)	14 (46.7%)		18 (46.2%)	37 (47.4%)	

Note

Between two stages, there was no significant difference between NPC and HCs in age and gender. Parameters were shown as number (percentage). Statistical contrast was exerted through the Wilcoxon‐Mann‐Whitney test.

### 3.2 Screening of candidate miRNAs

**FIGURE 1:**
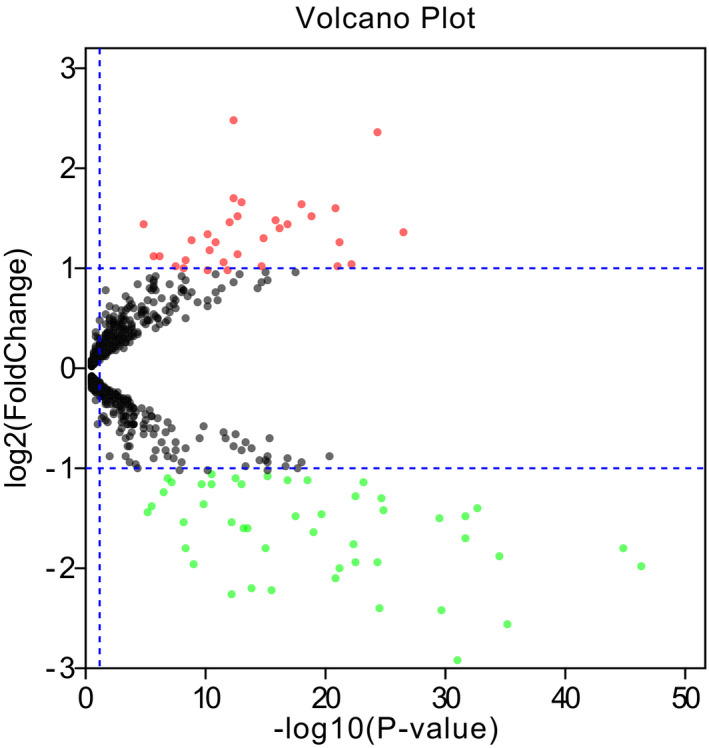
Volcano plot of the differentially expressed miRNAs in GSE32960

The gene expression data sets GSE32960 were downloaded from GEO (GPL14722 platform), with 312 NPC samples and 18 non‐cancer nasopharyngitis biopsy samples. The differentially expressed miRNAs were screened with adj.Pvalue < 0.05 and |logFC| > 1 by GEO2R (http://www.ncbi.nlm.nih.gov/geo/geo2r). Then, there were 32 up‐regulated miRNAs and 48 down‐regulated miRNAs (Figure 1). Table S2 lists the differently expressed miRNAs in GSE32960 with adj.Pvalue < 0.05 and |logFC| > 1. Combined with the review of the literature, we selected 10 miRNAs related to NPC as candidate miRNAs for further study.

### 3.3 Confirm candidate miRNAs during the testing phase

**FIGURE 2:**
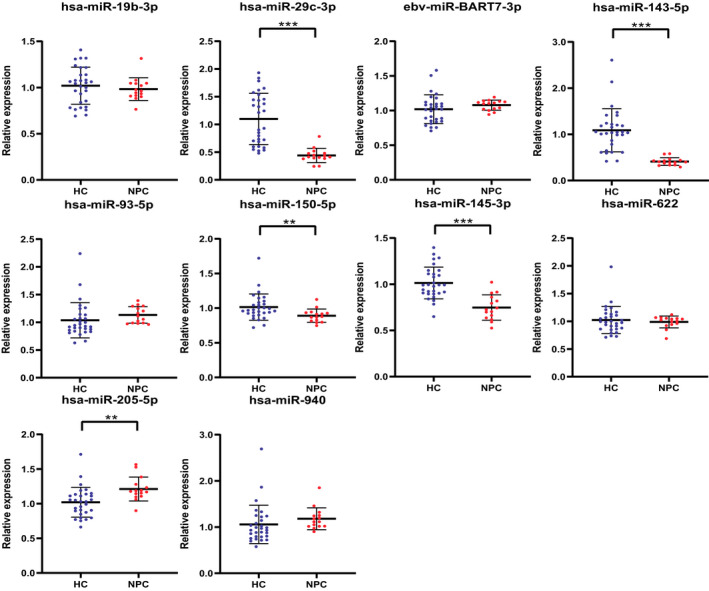
The expression levels of 10 candidate miRNAs in the testing phase. Screening conditions: *p*‐value < 0.05, * represents *p* < 0.05, ** represents *p* < 0.01, *** represents *p* < 0.001. The serum of 15 NPC patients and 30 HCs patients was used for testing phase

For the ten candidate miRNAs screened out, we verify them during the testing phase. There are randomly selected 15 cases of NPC patients and 30 cases of HCs. Through qRT‐PCR analysis, the results are shown in Figure 2. We can see that out of ten candidate miRNAs, there are still significant differences in expression between NPC patients and HCs in five of the ten candidate miRNAs (miR‐29c‐3p, miR‐143‐5p, miR‐150‐5p, miR‐145‐3p, and miR‐205‐5p). Therefore, we conducted further studies on these five miRNAs.

### 3.4 Detect the diagnostic value and expression level of these five miRNAs in the verification set

**FIGURE 3:**
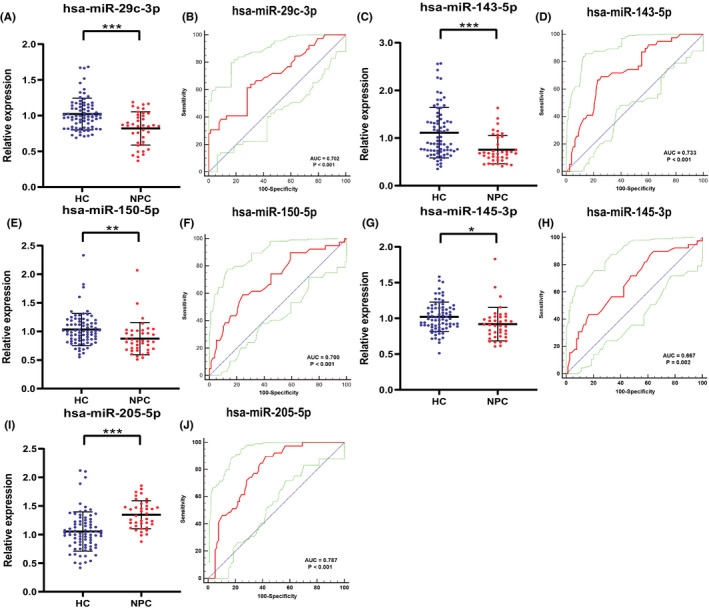
The relative expression of five miRNAs and the corresponding ROC curve in the verification stage. The sera of 39 NPC patients and 78 HCs patients were used for staging. The relative expression levels of (A) miR‐29c‐3p, (C) miR‐143‐5p, (E) miR‐150‐5p, and (G) miR‐145‐3p in the serum of NPC patients were significantly down‐regulated. (I) miR‐205‐5p is significantly up‐regulated. The area under the ROC curve is (B) miR‐29c‐3p with a 95% confidence interval of 0.611–0.783 is 0.702, (D) miR‐143‐5p with a 95% confidence interval of 0.644–0.811 is 0.733, (F) miR‐150‐5p with 95% confidence interval of 0.608–0.781 is 0.700, (H) miR‐145‐3p with 95% confidence interval of 0.574–0.752 is 0.667, and (J) miR‐205‐5p with 95% confidence interval of 0.701–0.857 is 0.787. * Represents *p* < 0.05, ** represents *p* < 0.01, *** represents *p* < 0.001. The red curve represents the receiver‐operating characteristic (ROC) curve. The green curve represents 95% ROC confidence interval. The blue line represents diagonal

We further studied these five miRNAs to verify whether their expression may be used as serum biomarkers in the screening of NPC. We selected 39 NPC patients and 78 HCs for further study. The results are shown in Figure 3. From the figure, we can know that the relative expression level of miR‐205‐5p is significantly increased in NPC patients, while the relative expression level of miR‐29c‐3p, miR‐143‐5p, miR‐150‐5p, miR‐145‐3p is decreased in NPC patients.

**FIGURE 4:**
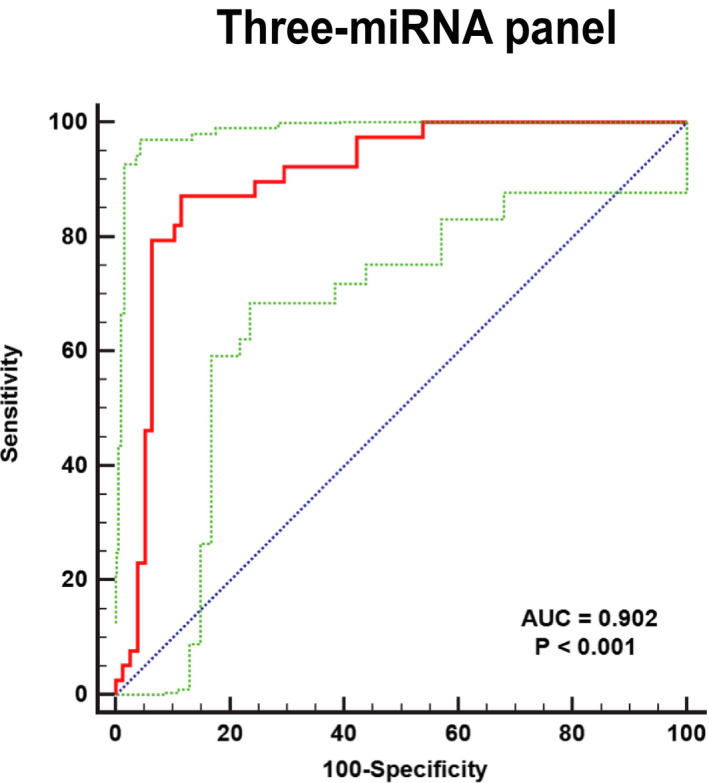
The receiver‐operating characteristic (ROC) curve analyses for the three‐miRNA panels. The AUC of the group consisting of three microribonucleic acids (miR‐29c‐3p, miR‐143‐5p, and miR‐205‐5p) is 0.903 (95% confidence interval: 0.833–0.949; sensitivity = 87.18%, specificity = 88.46%). The red curve represents the receiver‐operating characteristic (ROC) curve. The green curve represents 95% ROC confidence interval. The blue line represents diagonal

In order to evaluate the diagnostic value of these five miRNAs, we used the ROC curve for analysis. The AUCs were 0.702 (95% confidence interval [CI]: 0.611–0.783; Figure 3B) for miR‐29c‐3p, 0.733 (95% CI: 0.644–0.811; Figure 3D) for miR‐143‐5p, 0.700 (95% CI: 0.608–0.781; Figure 3F) for miR‐150‐5p, 0.667 (95% CI: 0.574–0.752; Figure 4H) for miR‐145‐3p, and 0.787 (95% CI: 0.701–0.857; Figure 3J) for miR‐205‐5p. Next, in Table 2, we use the Youden index to calculate the best cut‐off value and list the best specificity and sensitivity of these five miRNAs for diagnosing NPC. ROC curve analysis shows that miR‐29c‐3p, miR‐143‐5p, and miR‐205‐5p have moderate diagnostic capabilities for NPC (AUC are 0.702, 0.733, and 0.787, respectively).

**TABLE 2:** Outcomes of receiver‐operating characteristic curves and Youden index for five candidate miRNAs and the three‐miRNA panel

	AUC	*p* value	95% CI	Associated criterion	Sensitivity (%)	Specificity (%)
miR‐29c‐3p	0.702	<0.001	0.611–0.783	≤0.88	61.54	71.79
miR‐143‐5p	0.733	<0.001	0.644–0.811	≤0.71	66.67	76.92
miR‐150‐5p	0.700	<0.001	0.608–0.781	≤0.85	58.97	75.64
miR‐145‐3p	0.667	0.0019	0.574–0.752	≤0.84	43.59	83.33
miR‐205‐5p	0.787	<0.001	0.701–0.857	>1.06	89.74	57.69
Three‐miRNA panel	0.902	<0.001	0.833–0.949	>0.39012	87.18	88.46

Abbreviations: AUC, area under curve; CI, confidence interval.

### 3.5 Discover the best miRNA detection panel for NPC

In the verification stage, miR‐29c‐3p, miR‐205‐5p, and miR‐143‐5p have the moderate diagnostic ability for NPC. Since the combination of multiple miRNAs may be more accurate than a single miRNA, we built diagnostic panels. We combined miRNA expression data in the verification phase and found the best panel for diagnosing NPC among all combinations of three miRNAs through a stepwise logistic regression model. The model calculation formula is Logit(P) = 1.009 − 4.742 × miR‐29c‐3p − 2.393 × miR‐143‐5p + 3.994 × miR‐205‐5p. As shown in Figure 4, by drawing the ROC curve of the three miRNAs joint diagnostic panels, we found that its AUC is 0.902 (95% CI: 0.833–0.949; sensitivity = 87.18%, specificity = 88.46%; Table 2).

### 3.6 Bioinformatics analysis of candidate miRNAs

**FIGURE 5:**
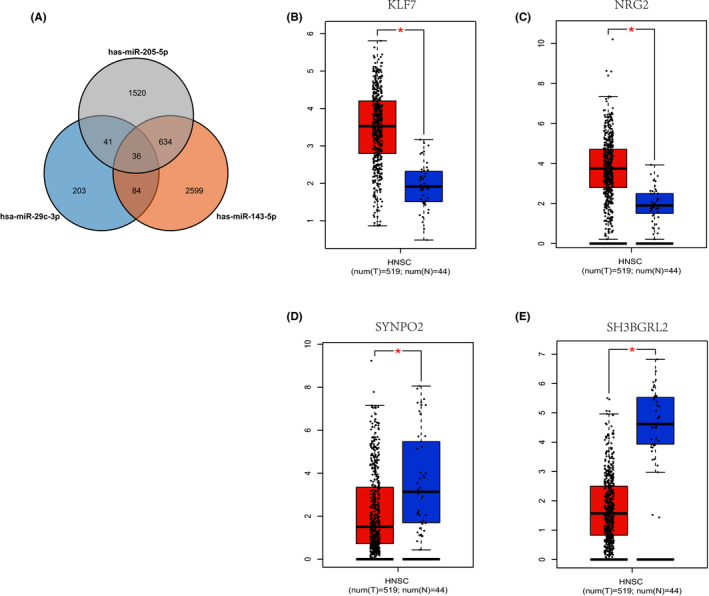
Target gene prediction and genes differentially expressed in GEPIA. (A) Using mirwalk target gene prediction and Enrichr annotation to predict the number of target genes: 795 (Genes predicted in more than two miRNAs were chosen as target genes). (B‐E) Among the 36 target genes predicted by all three miRNAs, searching in the GEPIA database, four genes have differences in HNSC expression (|log2FC| > 1.5, *p* < 0.01): KLF7(B), NRG1(C), SH3BGRL2(D), SYNPO2(E)

MiRWalk2.0 is used to predict the possible target genes of miR‐29c‐3p, miR‐143‐5p, and miR‐205‐5p. Select two or more miRNA‐predicted genes as target genes, and in total 283 genes were predicted (Figure 5A). Among the 36 target genes predicted by all three miRNAs, a search in the GEPIA database revealed that four genes had differences in Head and Neck squamous cell carcinoma (HNSC) expression.^14^ They are KLF7 (Figure 5B), NRG1 (Figure 5C), SH3BGRL2 (Figure 5D), SYNPO2 (Figure 5E), with log2FC > 1.5, *p* < 0.01, these four genes may be potential targets of the three‐miRNA diagnostic panels.

**FIGURE 6:**
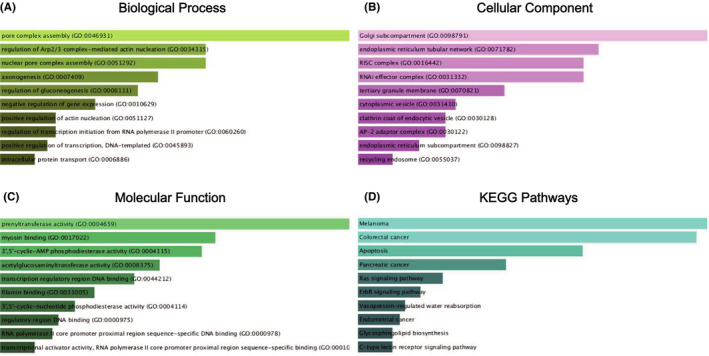
GO functional annotation and KEGG pathway enrichment analysis of the target genes of miR‐29c‐3p, miR‐143‐5p, and miR‐205‐5p. (A) Biological process (BP) analysis; (B) cellular component (CC) analysis; (C) molecular function (MF) analysis; (D) KEGG pathway enrichment analysis

In the next step, we use KEGG pathway enrichment analysis and GO function annotation to further study them in the Enrichr database. The KEGG pathway analysis of target genes shows that they are significantly enriched in Melanoma, Colorectal cancer, Apoptosis, and Pancreatic cancer (Figure 6D). The top 10 enriched GO items in each GO item in the GO function annotation are shown in Figure 6A–C. GO function annotation includes a biological process (BP), cell component (CC), and molecular function (MF). Including pore complex assembly (GO:0046931), regulation of Arp2/3 complex‐mediated actin nucleation (GO:0034315), and nuclear pore complex assembly (GO:0051292) in the BP category; RISC complex (GO:0016442), RNAi effector complex (GO:0031332), and endoplasmic reticulum tubular network (GO:0071782) in the CC category; prenyltransferase activity (GO:0004659), myosin binding (GO:0017022), and 3′,5′‐cyclic‐AMP phosphodiesterase activity (GO:0004115) in the MF category.

## 4 DISCUSSION

Studies have found that miRNAs are involved in cell metabolism pathways, cell‐to‐cell communication, and modification of the tumor microenvironment. Since the use of serum miRNA to screen for diseases is not only non‐invasive but also convenient, it has been widely used.^15^, ^16^ In this study, we designed a three‐stage test to find possible serum miRNAs for screening NPC. Through the three stages of screening, training, and verification, we finally found that there are five miRNAs (miR‐29c‐3p, miR‐143‐5p, miR‐150‐5p, miR‐145‐3p, and miR‐205‐5p) that are significantly different in the serum expression of NPC and HCs. Then, we constructed a three‐miRNA panel (AUC = 0.902; 95% CI: 0.833–0.949; sensitivity = 87.18%, specificity = 88.46%) containing miR‐29c‐3p, miR‐143‐5p, and miR‐205‐5p to screen for NPC and we analyze the clinical significance and potential function of each miRNA.

In this panel, the expression level of miR‐29c‐3p (AUC = 0.702; 95% confidence interval [CI]: 0.611–0.783; Figure 3B) is down‐regulated in NPC patients. New research has found that CD276 is a risk gene for multiple cancers. It can increase the phosphorylation level of Myc and inhibit the expression of miR‐29c‐3p. And the negative regulatory loop between CD276, Myc, and miR‐29c‐3p affects the cytotoxicity of cancer cells against NK cells.^17^ Previous studies have also shown that miR‐29c‐3p has been shown to be associated with gene expression in the development of nasopharyngeal carcinoma (NPC).^18^ Combining these studies with ours, miR‐29c‐3p may play an important role in intercellular molecular signaling pathways in the occurrence and development of NPC.

A large number of studies have shown that miR‐143‐5p is down‐regulated in a variety of tumors. It participates in cancer metastasis by targeting HIF‐1α/EMT‐related signaling pathways and is a potential therapeutic target.^19^ The study also found that HAGLR, as a competitive endogenous RNA of miR‐143‐5p, increases the expression of LAMP3, thereby promoting the proliferation, invasion, and migration of cancer cells.^20^ In this study, we first revealed the role of miR‐143‐5p in NPC screening (AUC = 0.733; 95% CI: 0.644–0.811; Figure 3D).

Studies have shown that the up‐regulation of miR‐205‐5p expression is closely related to the cell proliferation, cell migration, and clonogenic activity of HNSCC cells.^21^ Another study showed that miR‐205‐5p and its direct interaction with VENTXP1 regulate HNSCC cell proliferation and tumorigenicity. ANKRD2 has been identified as a miR‐205‐5p target and plays an important role in regulating NF‐kB signaling.^22^ Our research also shows that miR‐205‐5p is a good serum biomarker for NPC screening (AUC=0.787; 95% CI: 0.701–0.857; Figure 3J).

They are KLF7 (Figure 5B), NRG1 (Figure 5C), SH3BGRL2 (Figure 5D), SYNPO2 (Figure 5E), with log2FC > 1.5, *p* < 0.01, these four genes may be potential targets of the three‐miRNA diagnostic panels. Studies have shown that KLF7 is up‐regulated in gastric cancer. Overexpression of KLF7 promotes the proliferation and migration of gastric cancer cells. Mechanism analysis results show that KLF7 promotes the occurrence of gastric cancer by up‐regulating ANTXR cell adhesion molecule 1 (ANTXR1).^23^ In addition, a large number of studies have been conducted on NRG1 fusion as a carcinogenic driving factor for many tumor types, including nasopharyngeal carcinoma.^24^ SH3BGRL2 is also considered by various studies to play a role as a key tumor suppressor in the occurrence of cancers, such as glioblastoma and clear cell renal cell carcinoma.^25^, ^26^ SYNPO2, also known as synapsin 2 or myopod, encodes an actin‐binding protein and has been characterized as a tumor suppressor for aggressive cancers. According to reports, SYNPO2 can inhibit the activity of YAP/TAZ.^27^ In summary, these four genes play a role in the occurrence and development of a variety of cancers, so these genes may also have a close relationship with nasopharyngeal carcinoma. We will further explore the role of these genes in the occurrence and progression of nasopharyngeal carcinoma in future research.

There is no doubt that our research results are meaningful, but there are still some limitations. First of all, the number of patients included in this study is relatively small and the serum is taken from the patients before treatment, so we cannot know whether the levels of these miRNAs change after the patients receive treatment. In addition, although There are many miRNAs in the serum of nasopharyngeal cancer patients that are significantly different from HCs, in order to avoid possible negligence in data processing, we only included 10 miRNAs in this study. Therefore, in future research, we will continue to explore the value of other miRNAs in nasopharyngeal cancer screening and explore their biological effects.

In summary, this three‐miRNA panel has high diagnostic efficiency (AUC = 0.902;95% CI: 0.833–0.949; sensitivity = 87.18%, specificity = 88.46%; Table 2), and we believe that the three‐miRNA panel is likely to become a non‐invasive and novel biomarker for early screening and diagnosis of NPC. More studies are needed to confirm our findings.

## CONFLICT OF INTEREST

The authors declare that there are no conflicts of interest.

## AUTHOR CONTRIBUTIONS

Yongqing Lai and Hongyi Hu contributed to study concept and design. Rongkang Li and Chong Lu performed the experiments, and wrote the manuscript. Weiqiang Yang, Jiatao Zhong, and Yaqi Zhou collected the samples. Xuan Chen, Xinji Li, Guocheng Huang, Xiqi Peng, Kaihao Liu, and Chunduo Zhang performed the statistical analysis. All authors read and approved the final manuscript.

## Supporting information

Figure S1

Table S1

Table S2

Table S3

## Data Availability

The data that support the findings of this study are available from the corresponding author upon reasonable request.
