# Supplementary material for: A panel of three serum microRNA can be used as potential diagnostic biomarkers for nasopharyngeal carcinoma
Source: J Clin Lab Anal. 2022 Jan 14;36(2):e24194. doi: 10.1002/jcla.24194 (PMC8842135; doi:10.1002/jcla.24194)
Supplement: Supplementary file 2 — Table S1 [file JCLA-36-e24194-s004.docx]

**Table S1** Corresponding reference summary of 10 candidate miRNAs

| candidate miRNA | Corresponding reference title |
| --- | --- |
| hsa-miR-19b-3p | MicroRNA-19b-3p regulates nasopharyngeal carcinoma radiosensitivity by targeting TNFAIP3/NF-κB axis |
| hsa-miR-29c-3p | 1. Integrative Analysis Identified a 6-miRNA Prognostic Signature in Nasopharyngeal Carcinoma |
|  | 2. Meta-analysis of the differentially expressed microRNA profiles in nasopharyngeal carcinoma |
| ebv-miR-BART7-3p | 1. EBV-miR-BART7-3p Imposes Stemness in Nasopharyngeal Carcinoma Cells by Suppressing SMAD7 |
|  | 2. EBV-miR-BART7-3p promotes the EMT and metastasis of nasopharyngeal carcinoma cells by suppressing the tumor suppressor PTEN |
|  | 3. Gold nano-particles (AuNPs) carrying anti-EBV-miR-BART7-3p inhibit growth of EBV-positive nasopharyngeal carcinoma |
| hsa-miR-143-5p | 1.miR-143 inhibits proliferation and metastasis of nasopharyngeal carcinoma cells via targeting FMNL1 based on clinical and radiologic findings |
|  | 2.Functions of microRNA-143 in the apoptosis, invasion and migration of nasopharyngeal carcinoma |
|  | 3. Identification of miR-143 as a tumour suppressor in nasopharyngeal carcinoma based on microRNA expression profiling |
|  | 4. c-MYB regulates cell growth and DNA damage repair through modulating MiR-143 |
| hsa-miR-93-5p | 1. Integrative Analysis Identified a 6-miRNA Prognostic Signature in Nasopharyngeal Carcinoma |
|  | 2. Five miRNAs as novel diagnostic biomarker candidates for primary nasopharyngeal carcinoma |
| hsa-miR-150-5p | 1.miR‑150 inhibits proliferation and tumorigenicity via retarding G1/S phase transition in nasopharyngeal carcinoma |
|  | 2. miR-150 contributes to the radioresistance in nasopharyngeal carcinoma cells by targeting glycogen synthase kinase-3β |
|  | 3. MicroRNA profiling study reveals miR-150 in association with metastasis in nasopharyngeal carcinoma |
|  | 4. The Wnt modulator ICG‑001 mediates the inhibition of nasopharyngeal carcinoma cell migration in vitro via the miR‑150/CD44 axis |
| hsa-miR-145-3p | 1. Long noncoding RNA UCA1 promotes the proliferation, invasion, and migration of nasopharyngeal carcinoma cells via modulation of miR-145 |
|  | 2. Therapeutic targeting of CBP/β-catenin signaling reduces cancer stem-like population and synergistically suppresses growth of EBV-positive nasopharyngeal carcinoma cells with cisplatin  3. The long non-coding RNA MACC1-AS1 promotes nasopharyngeal carcinoma cell stemness via suppressing miR-145-mediated inhibition on SMAD2/MACC1-AS1 axis |
|  | 4. MicroRNA deregulation and pathway alterations in nasopharyngeal carcinoma |
| hsa-miR-622 | Differential miRNA expression and their target genes between NGX6-positive and negative colon cancer cells |
| hsa-miR-205-5p | 1.miR-205-5p regulates epithelial-mesenchymal transition by targeting PTEN via PI3K/AKT signaling pathway in cisplatin-resistant nasopharyngeal carcinoma cells |
|  | 2. Meta-analysis of the differentially expressed microRNA profiles in nasopharyngeal carcinoma |
|  | 3. Integrated analysis of microRNA regulatory network in nasopharyngeal carcinoma with deep sequencing |
|  | 4. miRNAs derived from circulating small extracellular vesicles as diagnostic biomarkers for nasopharyngeal carcinoma |
|  | 5. Identification of a 7-microRNA signature in plasma as promising biomarker for nasopharyngeal carcinoma detection |
| hsa-miR-940 | 1.Plasma microRNA expression signature involving miR-548q, miR-630 and miR-940 as biomarkers for nasopharyngeal carcinoma detection |
|  | 2. Depletion of intermediate filament protein Nestin, a target of microRNA-940, suppresses tumorigenesis by inducing spontaneous DNA damage accumulation in human nasopharyngeal carcinoma |
