# Supplementary material for: A panel of three serum microRNA can be used as potential diagnostic biomarkers for nasopharyngeal carcinoma
Source: J Clin Lab Anal. 2022 Jan 14;36(2):e24194. doi: 10.1002/jcla.24194 (PMC8842135; doi:10.1002/jcla.24194)
Supplement: Supplementary file 3 — Table S2 [file JCLA-36-e24194-s003.docx]

**Table S2** The differently expressed miRNAs in GSE32960 with adj.Pvalue <0.05 and |logFC|>1

|  | miRNA | logFC | P.Value | adj.P.Val |
| --- | --- | --- | --- | --- |
| upregulated | hsa-miR-767-5p | 2.50596 | 6.63E-14 | 8.10E-13 |
|  | ebv-miR-BART10 | 2.393 | 1.27E-26 | 7.29E-25 |
|  | ebv-miR-BART6-3p | 1.72709 | 6.71E-14 | 8.10E-13 |
|  | hsa-miR-622 | 1.68689 | 1.34E-14 | 1.84E-13 |
|  | ebv-miR-BART4 | 1.6732 | 6.44E-20 | 1.79E-18 |
|  | ebv-miR-BART16 | 1.62012 | 6.37E-23 | 2.25E-21 |
|  | ebv-miR-BART2-5p | 1.55708 | 2.73E-14 | 3.58E-13 |
|  | hsa-miR-194* | 1.54044 | 1.02E-20 | 3.02E-19 |
|  | hsa-miR-25* | 1.51479 | 1.03E-17 | 2.20E-16 |
|  | ebv-miR-BART7 | 1.49618 | 1.51E-13 | 1.76E-12 |
|  | hsa-miR-638 | 1.46476 | 4.97E-06 | 2.45E-05 |
|  | hsa-miR-93 | 1.46327 | 1.17E-18 | 2.75E-17 |
|  | hsa-miR-1238 | 1.43943 | 4.63E-18 | 1.01E-16 |
|  | hsa-miR-30c-2* | 1.38669 | 6.57E-29 | 5.02E-27 |
|  | ebv-miR-BART5 | 1.37831 | 1.30E-11 | 1.25E-10 |
|  | hsa-miR-658 | 1.33332 | 1.61E-16 | 2.73E-15 |
|  | hsa-miR-1915 | 1.31022 | 2.49E-10 | 2.22E-09 |
|  | ebv-miR-BART19-3p | 1.29334 | 2.45E-12 | 2.58E-11 |
|  | hsa-miR-663 | 1.2864 | 3.18E-23 | 1.21E-21 |
|  | ebv-miR-BART4-3p | 1.21167 | 7.28E-12 | 7.34E-11 |
|  | hsa-miR-1237 | 1.16286 | 2.66E-14 | 3.53E-13 |
|  | hsa-miR-205 | 1.15206 | 2.01E-07 | 1.28E-06 |
|  | hsa-miR-595 | 1.14567 | 7.86E-07 | 4.45E-06 |
|  | hsa-miR-30c-1* | 1.10556 | 1.19E-09 | 9.91E-09 |
|  | hsa-miR-760 | 1.09041 | 4.78E-13 | 5.34E-12 |
|  | hsa-miR-1323 | 1.07833 | 3.04E-24 | 1.27E-22 |
|  | hsa-miR-608 | 1.05492 | 2.78E-16 | 4.55E-15 |
|  | hsa-miR-99b* | 1.0488 | 4.14E-23 | 1.52E-21 |
|  | hsa-miR-1268 | 1.04649 | 8.24E-09 | 6.19E-08 |
|  | ebv-miR-BART6-5p | 1.03194 | 1.46E-09 | 1.21E-08 |
|  | hsa-miR-940 | 1.0094 | 2.04E-13 | 2.34E-12 |
|  | ebv-miR-BART9 | 1.00866 | 1.22E-11 | 1.19E-10 |
| downregulated | hsa-miR-146a | -1.0266 | 5.30E-12 | 5.47E-11 |
|  | hsa-miR-15a | -1.056 | 5.29E-17 | 1.06E-15 |
|  | hsa-miR-1260 | -1.0674 | 4.50E-08 | 3.04E-07 |
|  | hsa-miR-30a | -1.0749 | 3.92E-14 | 5.00E-13 |
|  | hsa-let-7e | -1.0819 | 1.22E-18 | 2.79E-17 |
|  | hsa-miR-19a | -1.0894 | 1.72E-20 | 4.94E-19 |
|  | hsa-miR-34b* | -1.1031 | 2.12E-25 | 1.08E-23 |
|  | hsa-miR-20a | -1.1083 | 2.03E-08 | 1.44E-07 |
|  | hsa-miR-491-3p | -1.1214 | 5.89E-12 | 6.00E-11 |
|  | hsa-miR-421 | -1.1222 | 1.06E-14 | 1.50E-13 |
|  | hsa-miR-199a-3p | -1.1348 | 4.36E-11 | 4.03E-10 |
|  | hsa-let-7b | -1.2056 | 7.32E-08 | 4.76E-07 |
|  | hsa-miR-126 | -1.2493 | 1.13E-24 | 5.19E-23 |
|  | hsa-miR-140-3p | -1.2782 | 5.60E-27 | 3.67E-25 |
|  | hsa-miR-23a | -1.3218 | 3.36E-11 | 3.15E-10 |
|  | hsa-miR-768-3p | -1.3458 | 9.04E-07 | 5.06E-06 |
|  | hsa-miR-374a | -1.3742 | 2.45E-35 | 3.74E-33 |
|  | hsa-miR-142-5p | -1.3956 | 3.83E-27 | 2.70E-25 |
|  | hsa-let-7a | -1.4033 | 2.18E-06 | 1.15E-05 |
|  | hsa-miR-22 | -1.4256 | 1.04E-21 | 3.30E-20 |
|  | hsa-miR-34c-5p | -1.4431 | 2.02E-19 | 5.15E-18 |
|  | hsa-miR-203 | -1.4436 | 3.19E-34 | 4.11E-32 |
|  | hsa-miR-342-3p | -1.4799 | 7.95E-32 | 6.63E-30 |
|  | hsa-miR-320b | -1.5144 | 1.80E-09 | 1.45E-08 |
|  | hsa-miR-99a | -1.5198 | 9.93E-14 | 1.17E-12 |
|  | hsa-miR-19b | -1.5724 | 9.88E-15 | 1.42E-13 |
|  | hsa-miR-451 | -1.574 | 3.43E-15 | 5.15E-14 |
|  | hsa-miR-145 | -1.6115 | 4.83E-21 | 1.48E-19 |
|  | hsa-miR-143 | -1.6602 | 3.58E-34 | 4.11E-32 |
|  | hsa-miR-30e | -1.7305 | 2.29E-24 | 1.00E-22 |
|  | hsa-miR-1181 | -1.7647 | 1.18E-09 | 9.91E-09 |
|  | hsa-miR-26b | -1.773 | 6.60E-48 | 3.03E-45 |
|  | hsa-miR-103 | -1.7794 | 1.06E-16 | 1.90E-15 |
|  | hsa-miR-125a-5p | -1.8599 | 3.63E-37 | 6.66E-35 |
|  | hsa-let-7g | -1.9024 | 1.04E-24 | 5.04E-23 |
|  | hsa-miR-100 | -1.9053 | 1.45E-26 | 7.84E-25 |
|  | hsa-miR-21 | -1.933 | 1.71E-10 | 1.54E-09 |
|  | hsa-miR-101 | -1.9407 | 8.97E-50 | 8.23E-47 |
|  | hsa-let-7d | -1.9757 | 3.01E-23 | 1.20E-21 |
|  | hsa-miR-29c | -2.0744 | 7.21E-23 | 2.45E-21 |
|  | hsa-miR-29a | -2.1627 | 1.64E-15 | 2.56E-14 |
|  | hsa-miR-26a | -2.1827 | 2.75E-17 | 5.74E-16 |
|  | hsa-miR-16 | -2.225 | 9.68E-14 | 1.15E-12 |
|  | hsa-let-7i | -2.3653 | 9.24E-27 | 5.65E-25 |
|  | hsa-let-7f | -2.381 | 3.86E-32 | 3.54E-30 |
|  | hsa-miR-29b | -2.5339 | 6.36E-38 | 1.46E-35 |
|  | hsa-miR-150 | -2.8864 | 1.61E-33 | 1.64E-31 |
|  | hsa-miR-142-3p | -3.3107 | 6.11E-47 | 1.87E-44 |
