# Supplementary material for: A panel of three serum microRNA can be used as potential diagnostic biomarkers for nasopharyngeal carcinoma
Source: J Clin Lab Anal. 2022 Jan 14;36(2):e24194. doi: 10.1002/jcla.24194 (PMC8842135; doi:10.1002/jcla.24194)
Supplement: Supplementary file 4 — Table S3 [file JCLA-36-e24194-s001.docx]

**Table S3** The primer sequences of these miRNAs.

| miRNA | Forward primer | Universal reverse primer |
| --- | --- | --- |
| hsa-miR-19b-3p | CGGTGTGCAAATCCATGCAA | CAGTGCAGGGTCCGAGGT |
| hsa-miR-29c-3p | GTCGGTAGCACCATTTGAAAT | CAGTGCAGGGTCCGAGGT |
| ebv-miR-BART7-3p | TGGCATCATAGTCCAGTGT | CAGTGCAGGGTCCGAGGT |
| hsa-miR-143-5p | TGGGTGCAGTGCTGCATC | CAGTGCAGGGTCCGAGGT |
| hsa-miR-93-5p | GGCAAAGTGCTGTTCGTGC | CAGTGCAGGGTCCGAGGT |
| hsa-miR-150-5p | CGGTCTCCCAACCCTTGTA | CAGTGCAGGGTCCGAGGT |
| hsa-miR-145-3p | TCGGGGATTCCTGGAAATAC | CAGTGCAGGGTCCGAGGT |
| hsa-miR-622 | TCGGACAGTCTGCTGAGGT | CAGTGCAGGGTCCGAGGT |
| hsa-miR-205-5p | TGGTCCTTCATTCCACCGG | CAGTGCAGGGTCCGAGGT |
| hsa-miR-940 | GGAGGAGCGGGGGCCC | CAGTGCAGGGTCCGAGGT |
| cel-miR-54-5p | TCGGAGGATATGAGACGACG | CAGTGCAGGGTCCGAGGT |
